# Reproductive factors and gall-bladder cancer, and the effect of common genetic variants on these associations: a case–control study in India

**DOI:** 10.1093/ije/dyab197

**Published:** 2021-09-22

**Authors:** Sharayu Mhatre, Ben Lacey, Paul Sherliker, Nilanjan Chatterjee, Preetha Rajaraman, Mahesh Goel, Shraddha Patkar, Vikas Ostwal, Prachi Patil, Shailesh V Shrikhande, Garvit Chitkara, Rajendra Badwe, Sarah Lewington, Rajesh Dikshit

**Affiliations:** Section of Molecular Epidemiology and Population Genetics, Centre for Cancer Epidemiology, Tata Memorial Centre, Kharghar, Navi Mumbai, India; Homi Bhabha National Institute (HBNI), Mumbai, India; Clinical Trial Service Unit and Epidemiological Studies Unit (CTSU), Nuffield Department of Population Health, University of Oxford, Oxford, UK; Clinical Trial Service Unit and Epidemiological Studies Unit (CTSU), Nuffield Department of Population Health, University of Oxford, Oxford, UK; MRC Population Health Research Unit, Nuffield Department of Population Health, University of Oxford, Oxford, UK; Division of Cancer Epidemiology and Genetics, National Cancer Institute, Bethesda, MD, USA; Department of Biostatistics, Bloomberg School of Public Health, John Hopkins University, Baltimore, MD, USA; Department of Oncology, School of Medicine, John Hopkins University, Baltimore, MD, USA; Office of Global Affairs, Department of Health and Human Services, Washington, DC, USA; Homi Bhabha National Institute (HBNI), Mumbai, India; Department of Surgical Oncology, Tata Memorial Hospital, Mumbai, Maharashtra, India; Homi Bhabha National Institute (HBNI), Mumbai, India; Department of Surgical Oncology, Tata Memorial Hospital, Mumbai, Maharashtra, India; Homi Bhabha National Institute (HBNI), Mumbai, India; Department of Medical Oncology, Tata Memorial Hospital, Mumbai, Maharashtra, India; Homi Bhabha National Institute (HBNI), Mumbai, India; Department of Medical Gastroenterology, Tata Memorial Hospital, Mumbai, Maharashtra, India; Homi Bhabha National Institute (HBNI), Mumbai, India; Department of Surgical Oncology, Tata Memorial Hospital, Mumbai, Maharashtra, India; Homi Bhabha National Institute (HBNI), Mumbai, India; Department of Surgical Oncology, Tata Memorial Hospital, Mumbai, Maharashtra, India; Homi Bhabha National Institute (HBNI), Mumbai, India; Department of Surgical Oncology, Tata Memorial Hospital, Mumbai, Maharashtra, India; Clinical Trial Service Unit and Epidemiological Studies Unit (CTSU), Nuffield Department of Population Health, University of Oxford, Oxford, UK; MRC Population Health Research Unit, Nuffield Department of Population Health, University of Oxford, Oxford, UK; UKM Medical Molecular Biology Institute (UMBI), Universiti Kebangsaan Malaysia, Kuala Lumpur, Malaysia; Section of Molecular Epidemiology and Population Genetics, Centre for Cancer Epidemiology, Tata Memorial Centre, Kharghar, Navi Mumbai, India; Homi Bhabha National Institute (HBNI), Mumbai, India

**Keywords:** Gall-bladder cancer, pregnancy, breastfeeding, menarche, menopause, case–control

## Abstract

**Background:**

In India, as elsewhere, the incidence of gall-bladder cancer (GBC) is substantially higher in women than in men. Yet, the relevance of reproductive factors to GBC remains poorly understood.

**Methods:**

We used logistic regression adjusted for age, education and area to examine associations between reproductive factors and GBC risk, using 790 cases of histologically confirmed GBC and group-matched 1726 visitor controls. We tested the interaction of these associations by genetic variants known to increase the risk of GBC.

**Results:**

Parity was strongly positively associated with GBC risk: each additional pregnancy was associated with an ∼25% higher risk {odds ratio [OR] 1.26 [95% confidence interval (95% CI) 1.17–1.37]}. After controlling for parity, GBC risk was weakly positively associated with later age of menarche [postmenopausal women, OR 1.11 (95% CI 1.00–1.22) per year], earlier menopause [OR 1.03 (95% CI 1.00–1.06) per year] and shorter reproductive lifespan [OR 1.04 (95% CI 1.01–1.07) per year], but there was little evidence of an association with breastfeeding duration or years since last pregnancy. Risk alleles of single-nucleotide polymorphisms in the *ABCB4 and ABCB1* genetic regions had a multiplicative effect on the association with parity, but did not interact with other reproductive factors.

**Conclusions:**

We observed higher GBC risk with higher parity and shorter reproductive lifespan, suggesting an important role for reproductive and hormonal factors.

Key Messages

- In this large case–control study among women in India, there was a strong linear association between risk of gall-bladder cancer (GBC) and parity throughout the range examined (up to ∼6 pregnancies—an analyses that would not have been possible in populations with lower birth rates), with each additional full-term pregnancy associated with an ∼25% higher risk.
- GBC risk was also associated with later menarche and earlier menopause, and as such shorter reproductive lifespan, but there was little evidence of an association with breastfeeding duration or years since last pregnancy.
- The effect of parity on GBC risk was further explored by assessing the joint effects of parity and common genetic variants known to confer higher risks of GBC. Risk alleles of single-nucleotide polymorphisms in the ABCB4 and ABCB1 genetic regions were observed to interact in a multiplicative manner on the association with parity, indicating a potential role for both reproductive and genetic factors in identifying those a particularly high risk of GBC who may benefit from screening.

## Background

In India, the incidence of gall-bladder cancer (GBC) is substantially higher in women than in men. In 2016, there were 26 000 incident cases of GBC in India, of which about two-thirds were in women; the incidence rate (age-standardized to Global Burden of Disease Study global reference population) was 3.3 per 100 000 in women and 1.9 per 100 000 among men.^1^ Substantial differences in incidence between the sexes is also seen in other parts of the world.^2^ As such, it has been hypothesized that reproductive and hormonal factors may have a causal role in GBC.

Some epidemiological studies evaluating the role of reproductive factors on GBC have suggested that parity, younger age at first birth and older age at menarche are associated with higher risk of GBC.^3–9^ However, these studies have been mainly conducted in high-income countries where parity is, on average, much lower than in low-income or middle-income countries, and the determinants of menarche and menopausal may be very different. Furthermore, the interaction between reproductive factors and genetics variants known to confer increased risk of GBC in the *ABCB4 and ABCB1* genetic regions that regulate hepatobiliary phospholipid transport [identified by GBC genome-wide association studies (GWAS)^10^] has not been evaluated.

We investigated the role of several reproductive factors in the development of GBC among pre- and postmenopausal women in a case–control study conducted in India. The study also aimed to examine the interaction of reproductive factors with GWAS-identified single-nucleotide polymorphisms (SNPs). To our knowledge, this is largest case–control study from a low-income country (where both parity and the risk of GBC are high) to evaluate the role of reproductive factors on the risk of GBC.

## Methods

### Study design and participants

Details of the study design have been described previously.^11^ In brief, we conducted a case–control study at the Tata Memorial Hospital (TMH), Mumbai, India, during the period 4 August 2015 to 17 May 2016 to investigate lifestyle and genetic risk factors for GBC. Men and women, aged ≥20 years, with histologically confirmed newly diagnosed GBC (ICD-O-3: site code C23) were enrolled as case subjects by trained social investigators. The TMC is a tertiary-level, specialist cancer centre that receives referrals from throughout India.

Visitors accompanying cancer patients at the TMH (for any cancer site except hepatobiliary malignancy) were recruited as control subjects; no single cancer site of the patients accompanying controls accounted for more than one-fifth of the total. Cases and controls were recruited simultaneously during the study period. The selection of controls was frequency-matched to cases based on age, sex and region of residence in India (north, north-east, west, central and south) at the time of diagnosis; and there was an approximately equal distribution between friends, neighbours, spouses and other relatives.

Data collected via questionnaire included: age, sex, region of residence, education, alcohol consumption, tobacco (smoking and chewing), medical history, current medication use and reproductive history (age at menarche, number of pregnancies, induced and spontaneous abortions, stillbirths, duration of breastfeeding with each child and age at menopause). Measurements of height, weight, waist and hip were also taken. For quality control, 10% of participants were reinterviewed, and study coordinators and data managers checked each questionnaire for implausible responses.

### Statistical analysis

Analyses were restricted to women and further excluded those with missing or outlying values of key variables (Supplementary Table S1, available as Supplementary data at *IJE* online). We estimated odds ratios (ORs) and corresponding 95% confidence intervals (CIs) for the association of GBC with reproductive factors by fitting unconditional logistic-regression models adjusted for age, education, region and menopausal status; further adjustment was made, where appropriate, for parity (number of full-term pregnancies) and total duration of breastfeeding (the sum of the period of breastfeeding for all children combined). Additional analyses were conducted by fitting these models after stratifying on menopausal status.

In categorical analyses, reproductive factors were grouped into the following categories: age at menarche (<14, 14, 15, ≥16 years), parity (0–2, 3, 4, ≥5), duration of breastfeeding (<44, 44-<72, 72-<102, 102-<138, ≥138 months), years since last pregnancy (0–7, 8–12, 13–17, ≥18 years), age at menopause (35–40, 41–45, 46–49, ≥50 years) and reproductive lifespan (<27, 27–30, 31–33, ≥34 years; defined as age at menopause minus age at menarche). For comparison of categories, the variance of the log odds in each group was calculated from the variances and covariances of the log OR; this provides group-specific CIs and appropriately attributes variance to all groups, including the reference, and so allows CIs to be used to compare risks in any two groups.^12^ Women whose menstrual periods had stopped either naturally or due to surgical intervention or any other reasons for ≥12 months from the date of enrolment were classified as postmenopausal. Sensitivity analyses were conducted by further adjusting for other potential confounding variables, including waist–hip ratio and history of gallstones.

To evaluate the interaction between reproductive factors and known genetic risk factors for GBC, we fitted logistic-regression models of reproductive variables stratified by common variants (AA allele vs AG/GG) of three SNPs: two in the *ABCB4* genetic region (rs1558375 and rs4148808) and one in the *ABCB1* genetic region (rs17209837). Analyses were adjusted for age, education, region, menopausal status and eigenvalues. We compared the difference in model deviance using a chi-squared test; the interaction was considered as significant if the *p*-value was <0.05. SAS software (v9.3) was used and graphs were plotted using R software (v3.0).

## Results

In total, 2513 women (790 cases and 1723 controls) were recruited into the study. Of these, 217 were excluded because they had missing or outlying values for reproductive factors, missing information on key covariates or were ever-smokers, leaving 692 GBC and 1604 controls (Supplementary Table S1, available as Supplementary data at *IJE* online).

Among controls, the mean age was 45 (SD 10) years, 915 (57%) were premenopausal and 376 (23%) had received <5 years of formal education. The mean age among cases was 49 (10) years, 231 (33%) were premenopausal and 338 (49%) had received <5 years of formal education. One hundred and two (15%) of the cases and 194 (12%) of the controls reported current tobacco chewing. Only two women reported ever drinking alcohol, but no information on alcohol consumption was available for 263 women. There was a substantially higher proportion of cases with a self-reported history of gallstones [310 (45%)] than controls [41 (3%)]. Almost all women reported one or more pregnancy (94%) and >95% of these women reported breastfeeding, although the parity and duration of breastfeeding were higher among cases than controls (Table 1).

**Table 1:** Characteristics of women with gall-bladder cancer (cases) and controls included in main analyses, menopausal status at recruitment

	Cases			Controls		
	Premenopausal	Postmenopausal	All	Premenopausal	Postmenopausal	All
Participants (*n*)	231	461	692	915	689	1604
Age (years)	39 (6)	54 (7)	49 (10)	38 (7)	54 (7)	45 (10)
Region of residence in India						
North	113 (38%)	213 (35%)	326 (36%)	192 (17%)	161 (19%)	353 (18%)
North-east	70 (23%)	154 (25%)	224 (24%)	236 (21%)	149 (18%)	385 (19%)
South	1 (0%)	1 (0%)	2 (0%)	14 (1%)	10 (1%)	24 (1%)
West	26 (9%)	64 (10%)	90 (10%)	426 (37%)	313 (37%)	739 (37%)
Central	21 (7%)	29 (5%)	50 (5%)	47 (4%)	56 (7%)	103 (5%)
Formal education completed						
<5 years	89 (39%)	249 (54%)	338 (49%)	188 (21%)	188 (27%)	376 (23%)
≥5 years	142 (47%)	212 (34%)	354 (39%)	727 (63%)	501 (60%)	1228 (62%)
Pregnancy-related factors						
Ever pregnant	225 (97%)	459 (100%)	684 (99%)	862 (94%)	676 (98%)	1538 (96%)
Women who breastfed	224 (97%)	453 (98%)	677 (98%)	840 (92%)	647 (94%)	1487 (93%)
Parity	3.2 (1.8)	4.4 (2.0)	4.0 (2.0)	2.4 (1.3)	3.1 (1.6)	2.7 (1.5)
Duration breastfeeding, years^a^	5.8 (3.8)	7.5 (4.5)	6.9 (4.3)	4.2 (3.2)	5.3 (4.0)	4.7 (3.6)
Years since last pregnancy	12 (7)	24 (8)	20 (9)	13 (7)	25 (8)	18 (10)
Age at menarche (years)	14.0 (1.3)	14.2 (1.6)	14.1 (1.5)	13.8 (1.5)	14.0 (1.5)	13.9 (1.5)
Age at menopause (years)	–	45.2 (4.8)	45.2 (4.8)	–	45.5 (5.1)	45.5 (5.1)
Waist–hip ratio^b^	0.86 (0.07)	0.88 (0.08)	0.87 (0.08)	0.83 (0.09)	0.85 (0.08)	0.84 (0.09)
History of gallstones^b^						
No	127 (55%)	252 (55%)	379 (55%)	896 (98%)	658 (96%)	1554 (97%)
Yes	102 (34%)	208 (34%)	310 (34%)	14 (1%)	27 (3%)	41 (2%)

Values are *N* (%) or mean (SD). Analyses exclude those with missing or outlying values for reproductive factors or key covariates, and those who were current or ex-smokers. Two women reported ever drinking alcohol (263 had missing data on alcohol consumption).

a All children combined.

b Information on waist–hip ratio missing for 35 women and on gallstone history for 12 women.

**Figure 1:**
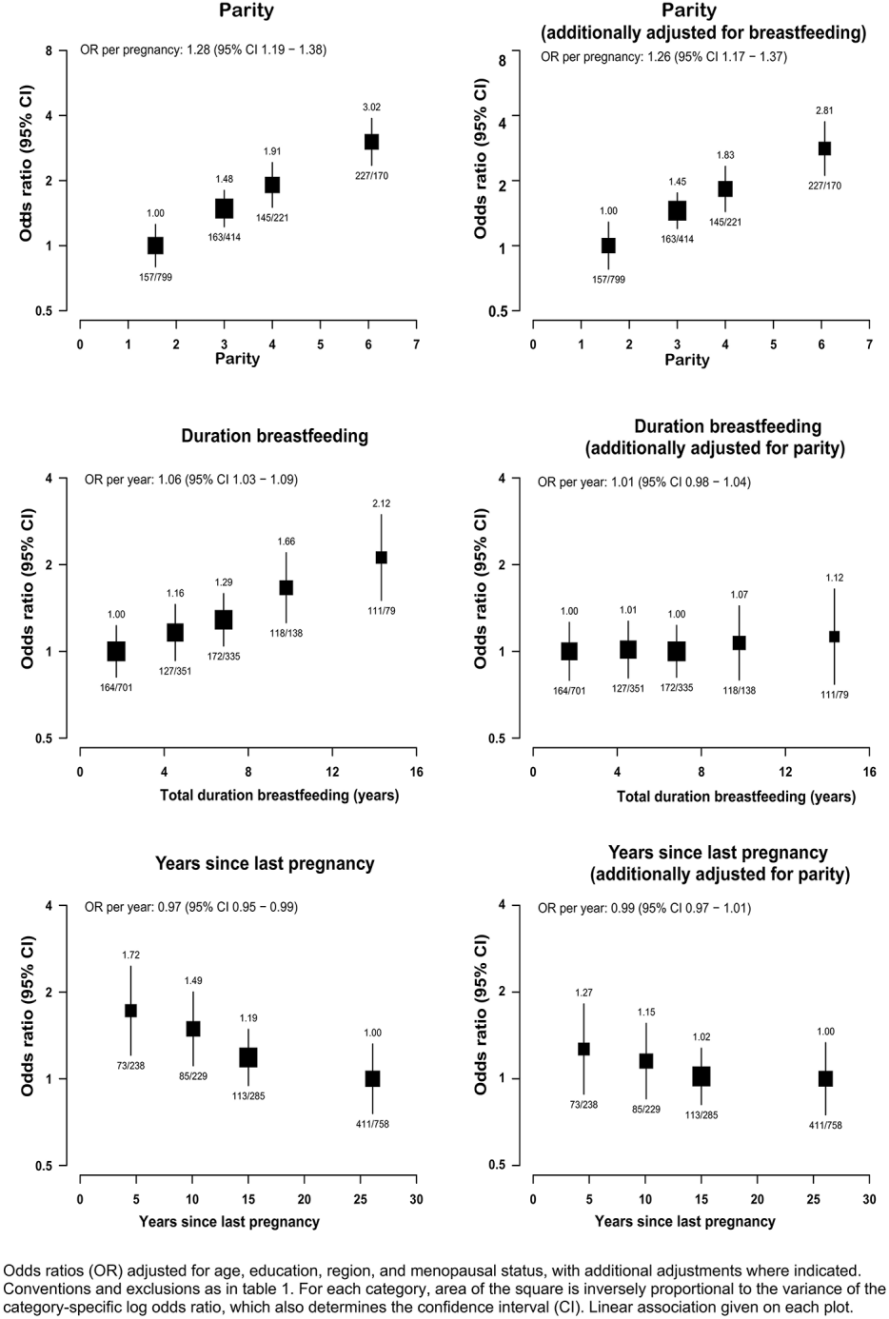
Association of gall-bladder-cancer risk with parity, duration of breastfeeding and years since last pregnancy

Parity was strongly positively associated with risk of GBC after adjusting for age, education, region and menopausal status, and was not materially changed by further adjustment for total duration of breastfeeding (Figure 1 and Supplementary Table S2, available as Supplementary data at *IJE* online). The association was approximately log-linear, with each additional pregnancy associated with an ∼25% higher risk of GBC [OR 1.26 (95% CI 1.17–1.37)]. There was no evidence of threshold even at high parity: six full-term pregnancies were associated with about three times the risk of GBC as two pregnancies. The association persisted even after adjustment for gallstones, and waist and hip circumferences (Supplementary Table S2, available as Supplementary data at *IJE* online).

**Figure 2:**
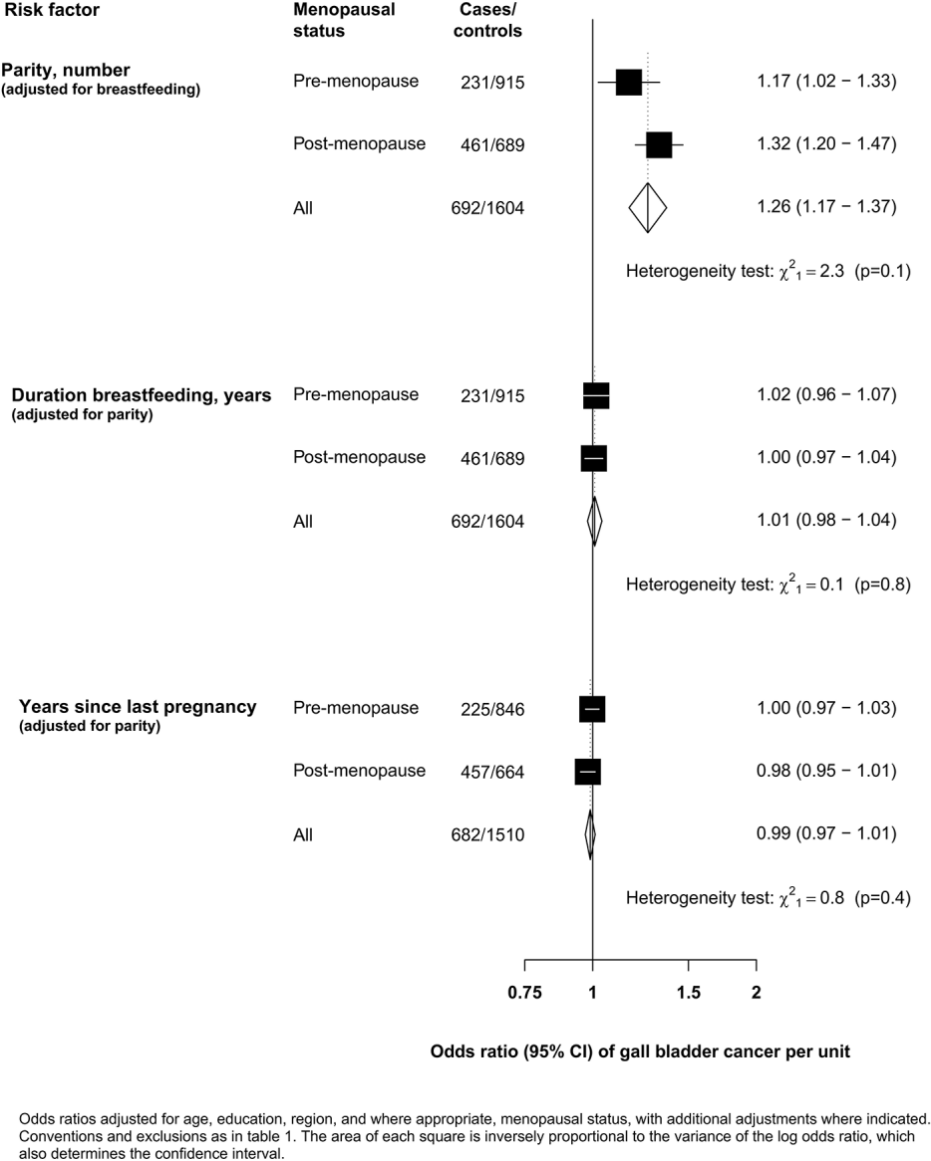
Association of gall-bladder-cancer risk with parity, duration of breastfeeding and years since last pregnancy by menopause status

Total duration of breastfeeding was also positively associated with GBC, but the association was almost completely attenuated following adjustment for full-term pregnancies [OR per year of breastfeeding 1.01 (95% CI 0.98–1.04)]. Similarly, the inverse association of GBC with years since last pregnancy was also largely attenuated following adjustment for full-term pregnancies [OR per year since last pregnancy 0.99 (95% CI 0.97–1.01)]. There was no evidence that the strength of the linear associations of GBC with any of the pregnancy-related factors differed by menopausal status (Figure 2).

**Figure 3:**
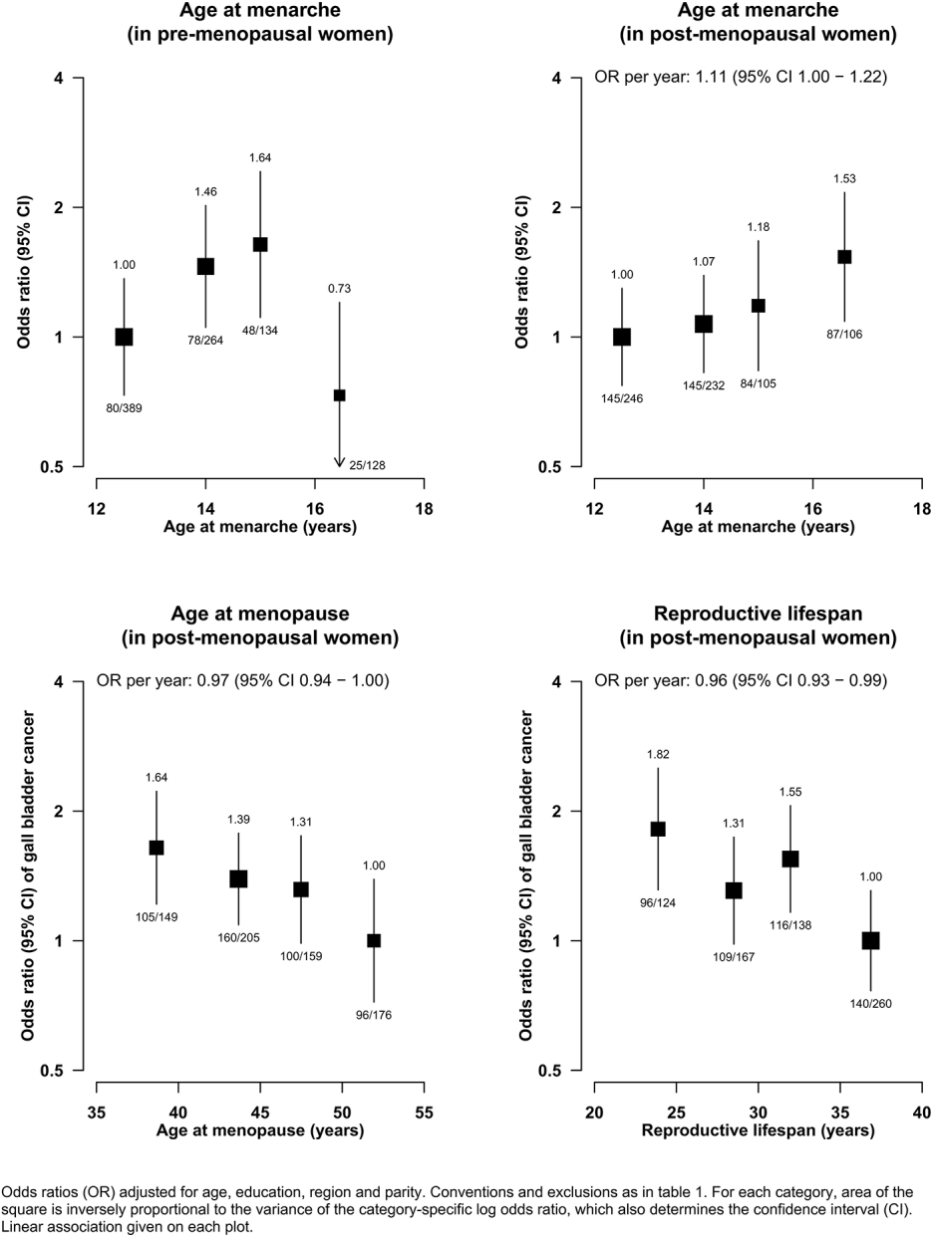
Association of gall-bladder-cancer risk with age at menarche, age at menopause and reproductive lifespan

By contrast, the association of GBC with age at menarche differed by menopausal status (Figure 3). Among postmenopausal women, there was a positive log-linear association of GBC with age at menarche [OR per year older age at menarche 1.11 (95% CI 1.00–1.22)], whereas in premenopausal women, there as a curvilinear association with lower risks of GBC at the extremes of the distribution of age at menarche. There was also evidence of a higher risk of GBC at younger age of menopause [OR per year younger 1.03 (95% CI 1.00–1.06)] and, as such, a shorter reproductive lifespan was associated with a higher risk of GBC [OR per year shorter 1.04 (95% CI 1.01–1.07)]; restricting these analyses to women with natural menopause did not materially change the strength of these associations (Supplementary Figure S1, available as Supplementary data at *IJE* online).

**Figure 4:**
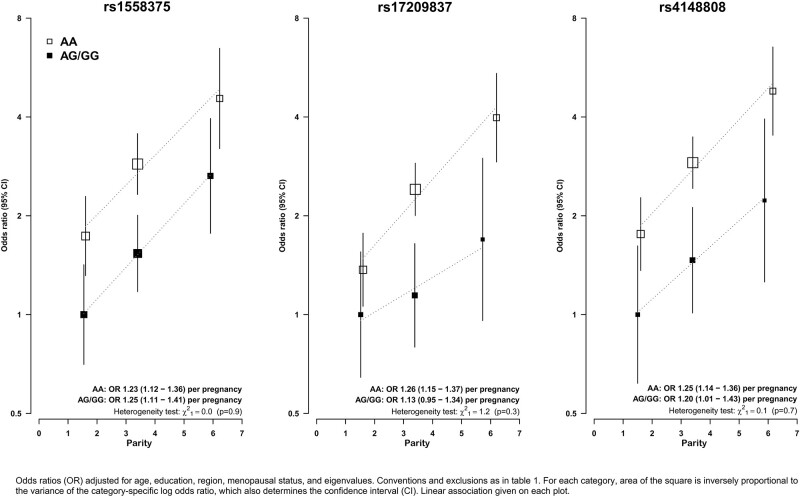
Association of gall-bladder-cancer risk with parity by genotype of single-nucleotide polymorphisms RS1558375, RS17209837 and RS4148808

The joint effect of parity with SNPs in the *ABCB4 and ABCB1* regions is shown in Figure 4 (see Supplementary Figure S2, available as Supplementary data at *IJE* online for plots on a linear scale). Homozygous alleles for SNPs rs1558375, rs17209837 and rs418808 were associated with a substantially higher risk of GBC, with OR for AA allele vs AG/GG of 1.57 (95% CI 1.23–2.00), 1.84 (95% CI 1.38–2.45) and 1.77 (95% CI 1.32–2.37), respectively. There was a multiplicative effect of each of these SNPs on the association with parity, such that women with AA allele and in the highest parity category (with, on average, approximately six pregnancies) had four times the risk of those with AG/GG allele in the lowest pregnancy category (with, on average, approximately two pregnancies). In sensitivity analyses (Supplementary Table S2, available as Supplementary data at *IJE* online), there was no evidence that any of the associations with reproductive factors were materially changed by further adjustment for waist–hip ratio or history of gallstones.

## Discussion

In this large case–control study, we evaluated the relationship between reproductive factors and GBC stratified by menopausal status. Parity was found to be strongly associated with GBC in both pre- and postmenopausal women. There was a log-linear association with each additional birth associated with an ∼25% higher GBC risk. After controlling for parity, GBC risk among postmenopausal women was weakly positively associated with later age of menarche and earlier menopause, such that a shorter reproductive lifespan was found to be associated with higher risk, but there was little evidence of an association with total breastfeeding duration or years since last pregnancy. Furthermore, risk alleles of SNPs in the *ABCB4 and ABCB1* genetic regions were observed to interact in a multiplicative manner with the association with parity.

There are few other large-scale studies of the association between parity and GBC risk. A large population-based case–control study conducted in Sweden (878 cases and 8238 controls) found a strong positive association of parity and GBC risk in postmenopausal, but not premenopausal, women: postmenopausal women with three or more children had greater than twice the risk [OR 2.34 (95% CI 1.87–2.93)] of uniparous postmenopausal women.^6^ A prospective study in Taiwan among 1.2 million parous women found that the risk of GBC mortality (257 deaths) increased by ∼20% with each additional birth, although there was substantial uncertainty about this estimate.^7^ A meta-analysis of prospective studies (including Asian and non-Asian women) reported a positive association between GBC and parity [hazard ratio per live birth 1.07 (95% CI 1.03–1.11)], albeit less steep than in the present report.^8^ Previous studies have also described positive associations of GBC risk with duration of breastfeeding and inverse associations with years since last pregnancy, but these studies have tended to be small and have not stratified findings by menopausal status or fully accounted for the correlation between these factors and parity.^3–5^^,^^9^^,^^13^

The associations of GBC with parity are biologically plausible and suggest a role for sex hormones in the aetiology of GBC. During pregnancy, elevated oestrogen levels result in an increase in the hepatic secretion of biliary cholesterol making bile supersaturated with cholesterol and lithogenic.^14^^,^^15^ Additionally, high levels of oestrogen and progesterone inhibit gall-bladder smooth-muscle contraction leading to gall-bladder stasis.^14^ These abnormalities promote the formation of biliary sludge and of gallstones during pregnancy,^13^ and gallstones and the associated inflammation are considered one of the major pathways in the carcinogenesis of GBC.^16^ Mendelian-randomization approaches using large-scale prospective studies with blood collected at baseline (‘biobanks’) might be particularly useful in assessing the causal pathways by which hormones may cause GBC. Several large biobanks are currently recruiting participants in India.

We observed a multiplicative effect of each of the previously identified GWAS SNPs in the *ABCB4 and ABCB1* genetic regions on the associations between parity and risk of GBC. These genes affect hepatobiliary phospholipids transport and were identified in GWAS studies to be associated with GBC.^10^ The *ABCB4* gene has been associated with intrahepatic cholestasis^17–20^ and polymorphism in these SNPs are associated with changes in the composition of bile, resulting in potent detergent and lithogenic properties.^21^ The multiplicative effect of these SNPs on the associations of GBC risk with parity indicates that these SNPs might usefully identify multiparous women at particularly high risk of GBC.

Results of previous studies on the association of breastfeeding with gallstones, cholecystitis and GBC have been equivocal. Breastfeeding has been associated with lower risk of gall-bladder disease in a large study of UK women (The Million Women study) and in a population-based study in China, Shanghai.^9^^,^^13^ With respect to GBC specifically, breastfeeding was associated with a higher risk of GBC in a South American Study^22^ but a lower risk in two studies in East Asia.^5^^,^^9^ The present study found no evidence of an association between breastfeeding and GBC risk, after accounting for parity, but given the very high proportion of women who breastfeed, it was not possible to assess reliably the relation between breastfeeding and not among parous women.

Consistently with some previous studies, we observed weak associations among postmenopausal women between a higher risk of GBC and later age at menarche and earlier age of menopause (i.e. shorter reproductive lifespan).^3–6^^,^^8^^,^^9^^,^^13^ There was some evidence of effect modification of the associations between GBC risk and age at menarche by menopausal status, but the reasons for this remain unclear. In particular, residual confounding cannot be excluded for the associations between reproductive lifespan and GBC risk, given the strength of these associations in the present report and the rapid economic development in India over the last few decades, which has been accompanied by marked changes in levels of both obesity and socio-economic status that are known to affect the age of menarche and age at menopause.^23^^,^^24^

The major strengths of this study include its size, the identification of incident cases of GBC and the standardized and detailed collection of data from trained field staff. All cases were histologically confirmed and, as such, misclassification of cancer types is unlikely. Furthermore, studies in high-income countries, where the average parity is substantially lower than in India, would not been able to assess reliably the effect of high levels of parity on GBC risk observed in the present study. It is a limitation of the study that information on exposure was not collected prior to disease occurrence to limit the effect of recall bias and that details on confounders prior to disease onset and at about the time of menarche and menopause (such as socio-economic status and any changes in place of residence) were not available to limit the effects of residual confounding; there was some evidence of slightly higher proportions of cases than controls with missing data for some variables (e.g. age and menarche and age at menopause), although the total exclusions for missing data were low. Also, the high rates of breastfeeding also meant it was not possible to assess reliably the effects of breastfeeding vs not breastfeeding among parous women in this population.

We did not adjust for gallstone disease in the main analyses as it is a potential mediator of the associations between reproductive factors and GBC risk,^25^ possibly through the effects of oestrogen on biliary cholesterol saturation.^15^ The prevalence of gallstone disease in India remains unclear, although a survey using ultrasound to diagnose gallstones in an area of northern India with high rates of GBC reported a prevalence among the adult population of ∼6%.^26^ In analyses of mediators in the present report (Supplementary Table S2, available as Supplementary data at *IJE* online), there was no material change in the risk estimates between reproductive factors and GBC risk after adjustment for history of gallstones, but ideally the assessment for prior gallstones would have included ultrasound screening of participants.

In conclusion, this is one of the largest studies to date to examine the effects of reproductive factors on the risk of GBC. We observed a strong positive association of GBC risk with parity and weaker associations with shorter reproductive lifespan, suggesting an important role for reproductive and hormonal factors in GBC. It is the first study to assess the joint effects of common genetic variants, known to confer higher risks of GBC, with parity. Given the multiplicative effect of SNPs in *ABCB4 and ABCB1* genetic regions on the associations of GBC with parity, the present study suggested that enhanced surveillance (such as by screening with abdominal ultrasound) among multiparous parous women with known genetic risk factors may be valuable in addressing the high rates of GBC among women.

## Supplementary data

Supplementary data are available at *IJE* online**.**

## Ethics approval

Ethical approval was by the Institutional Review Board of Tata Memorial Centre (TMH IRB Project Approval Number: 368).

## Funding

The Tata Memorial Centre, Department of Biotechnology [DBT-COE grant number BT/01CEIB/09/V/06]. B.L. acknowledges support from the UK Biobank, the National Institute for Health Research Biomedical Research Centre (Oxford, UK) and the BHF Centre of Research Excellence (Oxford, UK).

## Data availability

The data underlying this article will be shared on reasonable request to the corresponding author.

## Supplementary Material

dyab197_Supplementary_Data
