# Supplementary material for: Reproductive factors and gall-bladder cancer, and the effect of common genetic variants on these associations: a case–control study in India
Source: Int J Epidemiol. 2021 Sep 22;51(3):789–98. doi: 10.1093/ije/dyab197 (PMC9189936; doi:10.1093/ije/dyab197)
Supplement: dyab197_Supplementary_Data [file dyab197_supplementary_data.pdf]

**Reproductive factors and gallbladder cancer, and the effect of common genetic variants on these associations: a case-control study in India**

Supplementary Appendix, Table of Contents


| | Page |
|---|---|
| **Table S1:** Number of participants at baseline and exclusions from main analyses | 2 |
| **Table S2:** Association of gallbladder cancer risk with reproductive factors, with progressive adjustment for potential major confounders | 3 |
| **Figure S1:**Association of gallbladder cancer risk with age at menopause and reproductive lifespan, restricted to women known to have had natural menopause | 4 |
| **Figure S2:**Association of gallbladder cancer risk with parity, by genotype of single-nucleotide polymorphisms RS1558375, RS17209837, and RS4148808 (plotted on a linear scale) | 5 |

## Table S1: Number of participants at baseline and exclusions from main analyses

| | Overall, n(%) | Cases, n(%) | Controls, n(%) |
|---|---|---|---|
| **All participants** | 2513 (100) | 790 (100) | 1723 (100) |
| **Participants excluded due to:*** | | | |
| Missing ever pregnant | 20 (0.8) | 5 (0.6) | 15 (0.9) |
| Missing menopausal status | 1 (0.0) | 1 (0.1) | 0 (0.0) |
| Missing age at menarche | 55 (2.2) | 34 (4.3) | 21 (1.2) |
| Post-menopause, missing age at menopause | 19 (0.8) | 14 (1.8) | 5 (0.3) |
| Menopause age > current age | 4 (0.2) | 2 (0.3) | 2 (0.1) |
| Missing education | 5 (0.2) | 3 (0.4) | 2 (0.1) |
| Cancer diagnosis age > current age | 4 (0.2) | 0 (0.0) | 4 (0.2) |
| Ever-smoker or smoking missing | 26 (1.0) | 16 (2.0) | 10 (0.6) |
| Menopause before 35 | 72 (2.9) | 21 (2.7) | 51 (3.0) |
| Menopause at 60+ | 4 (0.2) | 2 (0.3) | 2 (0.1) |
| Menarche >18 | 7 (0.3) | 0 (0.0) | 7 (0.4) |
| Pregnancies, but breast feeding value missing | 0 (0.0) | 0 (0.0) | 0 (0.0) |
| **Total participants in main analyses** | 2296 (91.4) | 692 (87.6) | 1604 (93.1) |

*Number are for progressive exclusion of participants

**Table S2: Associations of reproductive factors and risk of gallbladder cancer, with progressive adjustment for potential major confounders**

| | | Odds ratio (95% CI) | | |
|---|---|---|---|---|
| | **Adjustments** | **Pre-menopause** | **Post-menopause** | **Overall** |
| **Full term pregnancies (per pregnancy)** | Age + Area | 1.37 (1.21 - 1.54) | 1.55 (1.41 - 1.69) | 1.48 (1.38 - 1.59) |
| | +education | 1.18 (1.03 - 1.33) | 1.34 (1.22 - 1.47) | 1.28 (1.19 - 1.38) |
| | +total breast feeding | 1.16 (1.02 - 1.32) | 1.32 (1.20 - 1.46) | 1.26 (1.16 - 1.36) |
| | +waist and hip circumferences* | 1.17 (1.02 - 1.34) | 1.33 (1.20 - 1.48) | 1.27 (1.17 - 1.38) |
| | +history of gall stones* | 1.11 (0.95 - 1.30) | 1.30 (1.15 - 1.46) | 1.22 (1.11 - 1.34) |
| **Duration of breastfeeding (per year)** | Age + Area | 1.13 (1.07 - 1.19) | 1.12 (1.08 - 1.16) | 1.12 (1.09 - 1.15) |
| | +education | 1.07 (1.02 - 1.13) | 1.06 (1.02 - 1.10) | 1.06 (1.03 - 1.09) |
| | +pregnancies | 1.02 (0.97 - 1.07) | 1.01 (0.97 - 1.04) | 1.01 (0.98 - 1.04) |
| | +waist and hip circumferences* | 1.02 (0.97 - 1.08) | 1.01 (0.97 - 1.05) | 1.01 (0.98 - 1.05) |
| | +history of gall stones* | 1.02 (0.96 - 1.09) | 1.01 (0.97 - 1.06) | 1.02 (0.98 - 1.06) |
| **Years since last pregnancy (per year)** | Age + Area | 0.98 (0.95 - 1.00) | 0.96 (0.93 - 0.99) | 0.97 (0.95 - 0.99) |
| | +education | 0.98 (0.95 - 1.01) | 0.97 (0.94 - 1.00) | 0.97 (0.95 - 0.99) |
| | +pregnancies | 1.00 (0.97 - 1.03) | 0.98 (0.95 - 1.01) | 0.99 (0.97 - 1.01) |
| | +waist and hip circumferences* | 1.00 (0.97 - 1.03) | 0.98 (0.95 - 1.01) | 0.99 (0.97 - 1.01) |
| | +history of gall stones* | 1.00 (0.96 - 1.03) | 0.98 (0.95 - 1.02) | 0.99 (0.97 - 1.02) |
| **Age at menarche (per year)** | Age + Area | 1.02 (0.90 - 1.16) | 1.13 (1.02 - 1.24) | 1.09 (1.01 - 1.17) |
| | +education | 1.01 (0.88 - 1.16) | 1.11 (1.01 - 1.23) | 1.08 (0.99 - 1.17) |
| | +pregnancies | 1.00 (0.87 - 1.15) | 1.10 (1.00 - 1.22) | 1.07 (0.98 - 1.16) |
| | +waist and hip circumferences* | 1.01 (0.87 - 1.16) | 1.11 (1.00 - 1.23) | 1.07 (0.98 - 1.17) |
| | +history of gall stones* | 1.02 (0.86 - 1.20) | 1.11 (0.98 - 1.26) | 1.08 (0.98 - 1.19) |
| **Age at menopause (per year)** | Age + Area | - | 0.95 (0.92 - 0.98) | - |
| | +education | - | 0.96 (0.93 - 0.99) | - |
| | +pregnancies | - | 0.97 (0.94 - 1.00) | - |
| | +waist and hip circumferences* | - | 0.97 (0.94 - 1.00) | - |
| | +history of gall stones* | - | 0.97 (0.94 - 1.01) | - |
| **Reproductive lifespan (per year)** | Age + Area | - | 0.95 (0.92 - 0.98) | - |
| | +education | - | 0.96 (0.93 - 0.99) | - |
| | +pregnancies | - | 0.96 (0.93 - 0.99) | - |
| | +waist and hip circumferences* | - | 0.97 (0.94 - 1.00) | - |
| | +history of gall stones* | - | 0.97 (0.93 - 1.00) | - |

Conventions and exclusions as per table 1. *Information on waist-hip ratio missing for 35 women, and on gallstone history for 12 women (n.b. gallstones may also mediate the association with between reproductive factors and gallbladder cancer).

**Figure S1: Association of gallbladder cancer risk with age at menopause and reproductive lifespan, restricted to women known to have had natural menopause**

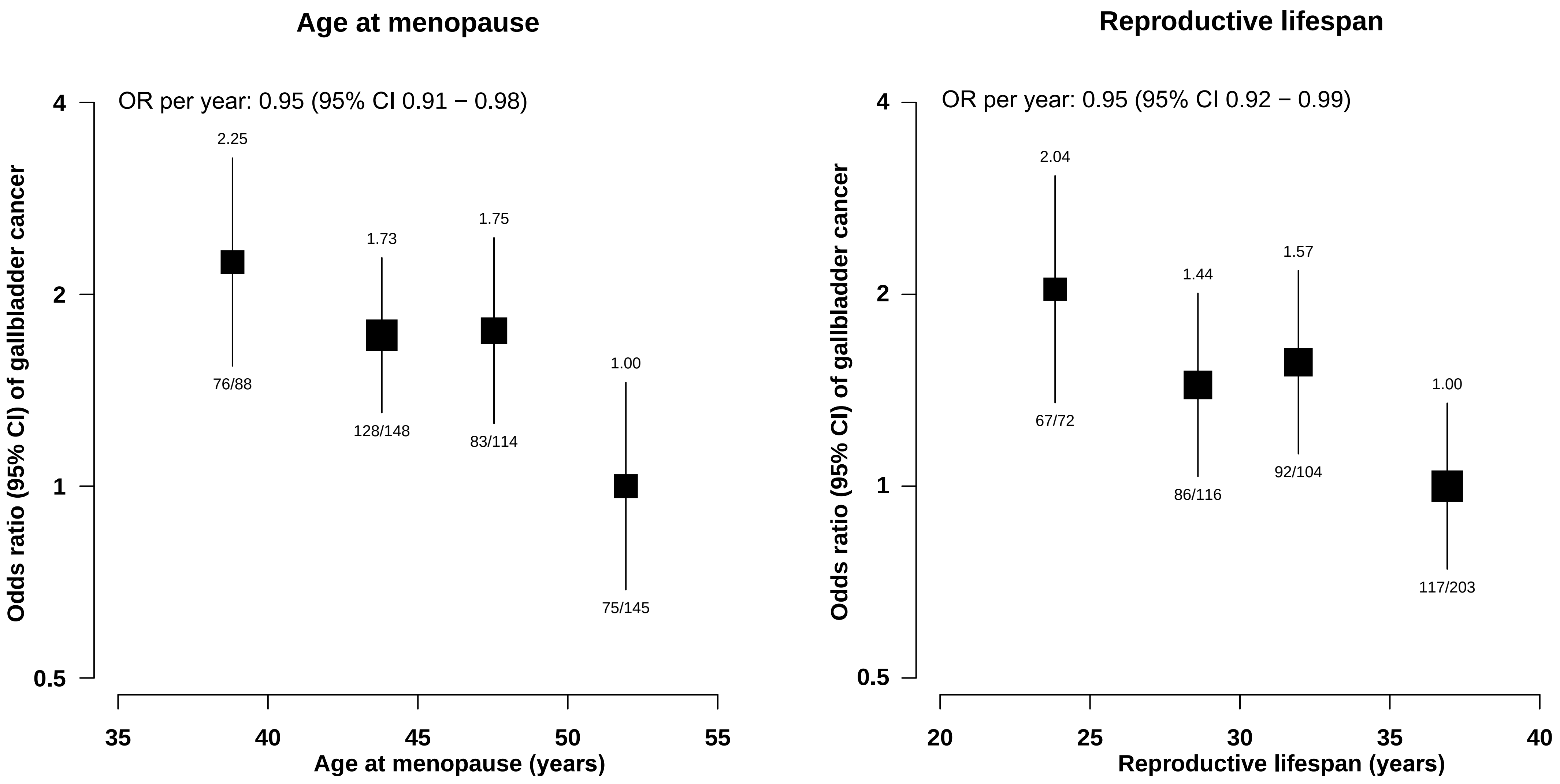


Odds ratios (OR) adjusted for age, education, region, and parity. Analyses restricted to women known to have had natural menopause, with further exclusions and conventions as in table 1. For each category, area of the square is inversely proportional to the variance of the category-specific log odds ratio, which also determines the confidence interval (CI). Linear associations given on each plot.

## Figure S2: Association of gallbladder cancer risk with parity, by genotype of single-nucleotide polymorphisms RS1558375, RS17209837, and RS4148808 (plotted on a linear scale)

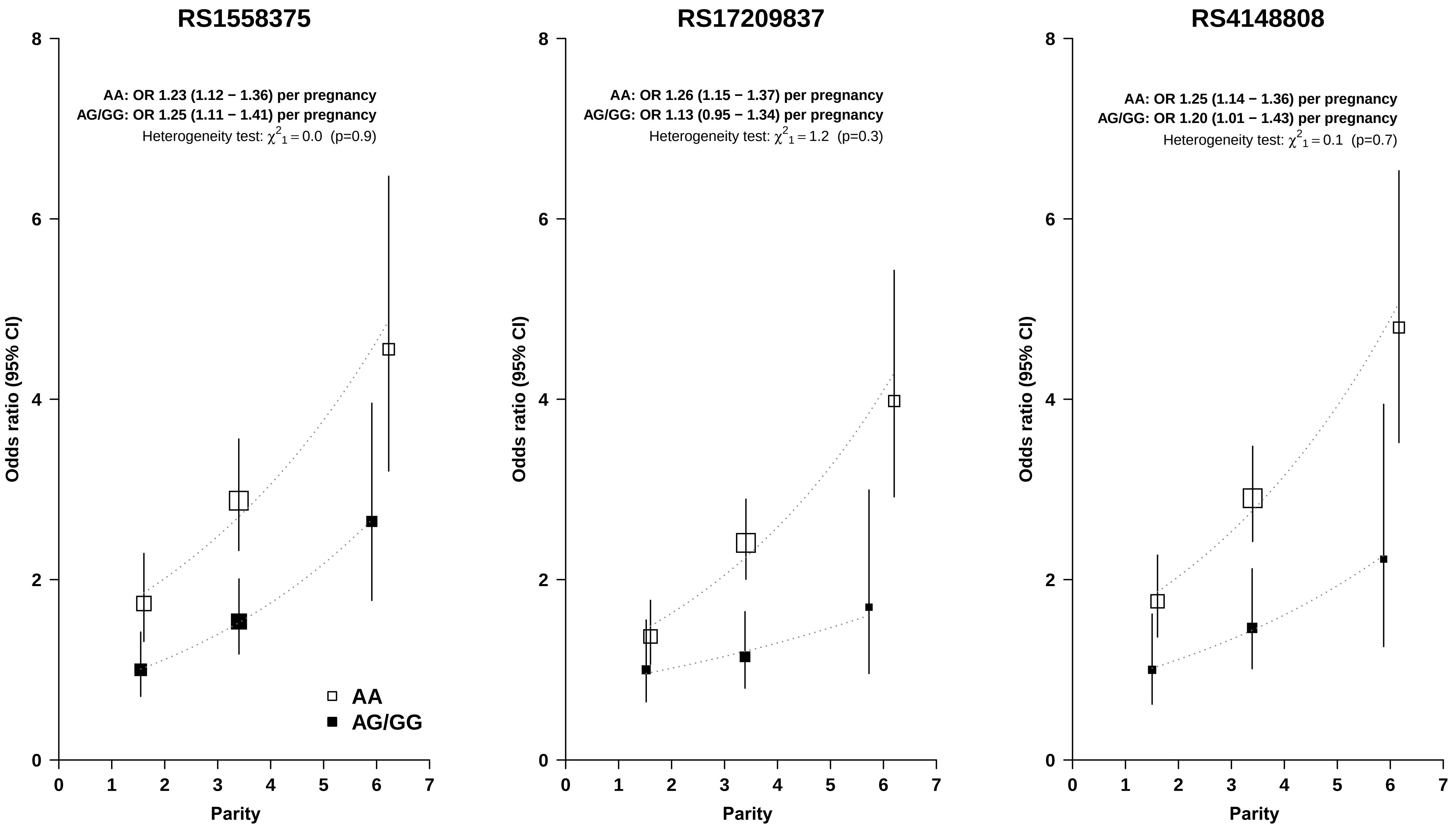


Odds ratios (OR) adjusted for age, education, region, menopausal status, and eigenvalues. Conventions and exclusions as in table 1. For each category, area of the square is inversely proportional to the variance of the category-specific log odds ratio, which also determines the confidence interval (CI). Linear associations given on each plot.
